# Body Composition in Cases with Normal Alanine Aminotransferase Values in Medical Health Checkups

**DOI:** 10.3390/nu16223847

**Published:** 2024-11-10

**Authors:** Kosuke Ushiro, Akira Fukuda, Masahiro Matsui, Saori Onishi, Tomohiro Nishikawa, Akira Asai, Soo Ki Kim, Hiroki Nishikawa

**Affiliations:** 1 Second Department of Internal Medicine, Osaka Medical and Pharmaceutical University, Takatsuki 569-8686, Osaka, Japan; 2 Health Science Clinic, Osaka Medical and Pharmaceutical University, Takatsuki 569-8686, Osaka, Japan; 3 Department of Gastroenterology, Kobe Asahi Hospital, Kobe 653-8501, Hyogo, Japan

**Keywords:** ALT, fat mass, fat-free mass, fat mass to fat-free mass ratio, sarcopenia

## Abstract

Background and aims: We aimed to clarify the relationship between alanine aminotransferase (ALT) level and body composition in Japanese medical health checkups, especially in cases with ALT ≤ 30 IU/L (7569 men and 9497 women). Methods: We categorized our study cohort into four groups: type A (ALT value ≤ 10 IU/L), type B (11 ≤ ALT value ≤ 20 IU/L), type C (21 ≤ ALT value ≤ 30 IU/L) and type D (ALT value > 30 IU/L (ALT over 30)). We retrospectively compared body composition-related parameters (body mass index (BMI), waist circumference (WC), fat (F) index, fatty liver index (FLI), fat-free (FF) index and F-FF ratio) among the four types. Results: Type A/B/C/D in men and women was found in 262/3279/2107/1921 and 1549/5736/1495/717 (*p* < 0.0001). BMI, WC, F-index, FLI, FF index and F-FF ratio were all significantly stratified among the four types, regardless of whether they were male or female and over or under 50 years old. Conclusions: With a decrease in ALT level in medical health checkups, fat mass decreases, and F-FF ratio decreases, but a decrease in skeletal muscle mass cannot be overlooked.

## 1. Introduction

The “Nara Declaration” was issued by the Japan Society of Hepatology in 2023 [1]. The purpose of this declaration is to promote early detection and early treatment of liver diseases through cooperation between family doctors and specialists using alanine aminotransferase (ALT) values as an indicator. In cases with ALT > 30 IU/L in a physical examination, etc., the patient should first visit his/her family doctor, etc., who will search for the cause of the problem and, if necessary, the patient will undergo a thorough examination at a specialized department such as a gastroenterology clinic [1]. On the other hand, cases with ALT ≤ 30 IU/L are likely to be followed up without undergoing any specific scrutiny. However, there is a wide range of cases of ALT ≤ 30 IU/L, from ALT ≤ 10 IU/L to ALT close to 30 IU/L. In our previous report, we reported a significant improvement in the balance between fat and muscle mass when ALT decreased from >30 IU/L to ≤30 IU/L [2]. However, the details of body composition in cases with ALT ≤ 30 IU/L were not available in that study.

In view of study reports on ALT and body composition, the following has been reported: ALT is correlated well with body fat mass and lean mass in women [3]. In overweight or obese individuals, ALT is correlated positively with body fat mass and lean body mass in men and lean body mass in women [4]. In children, elevated ALT is correlated closely with metabolic syndrome [5]. ALT was found to be positively correlated with both fat mass and lean mass in young adults [6]. In the present study, in order to clarify the relationship between ALT level and body composition in Japanese medical health checkups, especially in cases with ALT ≤ 30 IU/L, we classified cases with ALT ≤ 30 IU/L into three groups according to the ALT level and examined the relationship between ALT and body composition, comparing with cases of ALT over 30 IU/L. In other words, body composition was compared among the four groups, but such reports are rare.

## 2. Patients and Methods

### 2.1. Patients and Our Study

Between May 2023 and June 2024, a total of 17,066 consecutive medical health checkups with data on somatic composition were found in our medical database and were analyzed retrospectively. All study subjects were tested at the Osaka Medical and Pharmaceutical University (OMPU) Health Sciences Clinic (OMPU-attached facility). Our method of measuring the somatic composition is described elsewhere [7]. We have used TANITA (body composition analyzer with automatic height meter, DC-270A-N, Tokyo, Japan) for the assessment of body composition [7]. Fat (F) mass and fat-free (FF) mass were measured in the current analysis. The F index was defined as fat mass divided by height squared (kg/m^2^). The FF index was defined as fat-free mass divided by height squared (kg/m^2^). The Fat mass to fat-free mass (F-FF) ratio was defined as the F index divided by the FF index. In accordance with a previous report, skeletal muscle mass (SMM) loss was defined as an FF index < 18 kg/m^2^ in men and an FF index < 15 kg/m^2^ in women [8]. The fatty liver index (FLI) was calculated as reported elsewhere [9].

This study conformed to the principles of the Declaration of Helsinki and was approved by the ethics committee of OMPU hospital (approval no. 2024-135). An opt-out approach was used to obtain informed consent from study subjects, and personally identifiable information was completely protected during data collection.

### 2.2. Our Type Classification

Type A was defined as having an ALT value ≤ 10 IU/L. Type B was defined as 11 IU/L ≤ ALT value ≤ 20 IU/L. Type C was defined as 21 IU/L ≤ ALT value ≤ 30 IU/L. Type D was defined as having an ALT value > 30 IU/L (ALT over 30). We retrospectively compared body composition-related parameters (body mass index (BMI), waist circumference (WC), F index, FLI, FF index and F-FF ratio) among types A, B, C and D.

### 2.3. Statistics

In two-group comparisons (continuous variables), an unpaired *t*-test or Mann–Whitney *U*-test was used, as appropriate, after verifying the equal variance. In multiple-group comparisons (continuous variables), analysis of variance (ANOVA) or the Kruskal–Wallis test was used, as appropriate, after verifying the equal variance. Fisher’s exact test was used in the group comparisons (nominal variables). The clinical data were shown as number (*n*) or median (interquartile range (IQR)) with a *p* value < 0.05 as statistically significant using JMP 17.2.0 software (SAS Institute, Cary, NC, USA) for statistics.

## 3. Results

### 3.1. Baseline Features

Baseline features in the present research are shown in Table 1. Type A/B/C/D in men and women was found in 262 (3.5%)/3279 (43.3%)/2107 (27.8%)/1921 (25.4%) and 1549 (16.3%)/5736 (60.4%)/1495 (15.7%)/717 (7.6%) (*p* < 0.0001). The median (IQR) FLI in men and women was 24.78 (10.36–49.24) and 6.56 (3.11–17.23) (*p* < 0.0001). The median (IQR) F index in men and women was 5.06 (3.83–6.45) and 6.03 (4.67–7.94) kg/m^2^ (*p* < 0.0001). The median (IQR) FF index in men and women was 18.38 (17.48–19.38) and 15.11 (14.48–15.77) kg/m^2^ (*p* < 0.0001). The median (IQR) F-FF ratio in men and women was 0.28 (0.22–0.34) and 0.40 (0.32–0.51) (*p* < 0.0001).

### 3.2. Body Composition-Related Parameters Among Four Types in Men and Women

**Figure 1:**
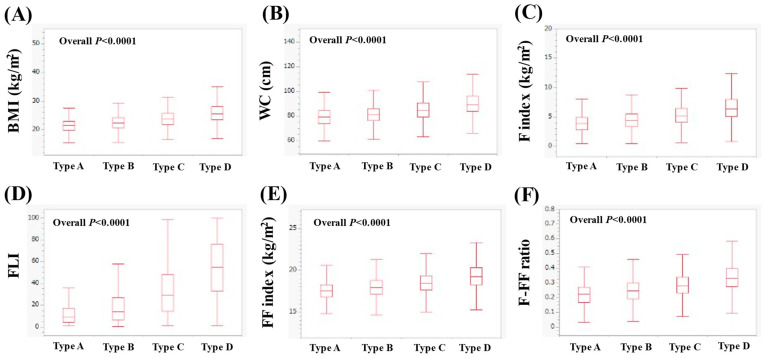
Comparison of body composition-related parameters among types A (*n* = 262), B (*n* = 3279), C (*n* = 2107) and D (*n* = 1921) in men. (**A**) BMI, (**B**) WC, (**C**) F index, (**D**) FLI, (**E**) FF index and (**F**) F-FF ratio.

In men, the median (IQR) BMI in types A, B, C and D was 21.5 (19.8–23.0), 22.3 (20.7–24.2), 23.7 (21.9–25.7), and 25.6 (23.5–28.1) kg/m^2^ (*p* < 0.0001 for all two-group comparisons, overall *p* < 0.0001, Figure 1A). The median WC (IQR) in types A, B, C and D was 79 (73.5–84.1), 81 (76–86), 84.5 (79–90.5) and 89.5 (84–96) cm (*p* < 0.0001 for all two-group comparisons, overall *p* < 0.0001, Figure 1B). The median (IQR) F index in types of A, B, C and D was 3.93 (2.82–4.94), 4.39 (3.37–5.57), 5.22 (4.09–6.42) and 6.35 (5.11–8.02) kg/m^2^ (*p* < 0.0001 for all two-group comparisons, overall *p* < 0.0001, Figure 1C). The median (IQR) FLI in types A, B, C and D was 8.92 (4.14–17.53), 14.11 (6.79–27.17), 29.15 (14.48–48.19) and 54.8 (32.97–76.06) (*p* < 0.0001 for all two-group comparisons, overall *p* < 0.0001, Figure 1D). The median (IQR) FF index in types A, B, C and D was 17.57 (16.80–18.30), 17.99 (17.16–18.82), 18.49 (17.62–19.39) and 19.26 (18.26–20.30) kg/m^2^ (*p* < 0.0001 for all two-group comparisons, overall *p* < 0.0001, Figure 1E). The median (IQR) F-FF ratio in types A, B, C and D was 0.23 (0.17–0.27), 0.25 (0.19–0.30), 0.28 (0.23–0.34) and 0.33 (0.28–0.40) (*p* < 0.0001 for all two-group comparisons, overall *p* < 0.0001, Figure 1F).

**Figure 2:**
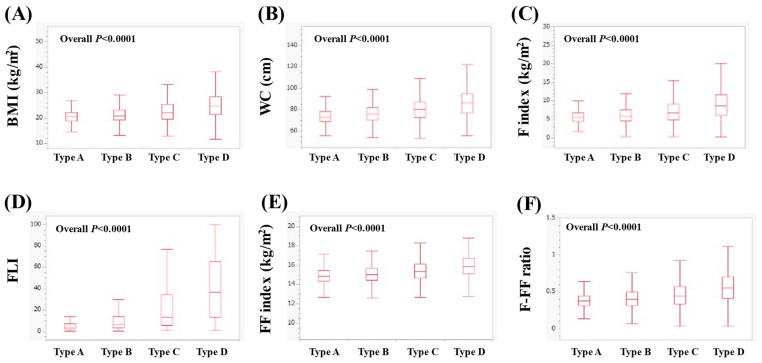
Comparison of body composition-related parameters among types A (*n* = 1549), B (*n* = 5736), C (*n* = 1495) and D (*n* = 717) in women. (**A**) BMI, (**B**) WC, (**C**) F index, (**D**) FLI, (**E**) FF index and (**F**) F-FF ratio.

In women, the median (IQR) BMI in types A, B, C and D was 20.4 (18.9–22.1), 20.8 (19.1–23.1), 22.1 (19.6–25.3) and 24.7 (21.3–28.45) kg/m^2^ (*p* < 0.0001 for all two-group comparisons, overall *p* < 0.0001, Figure 2A). The median WC (IQR) in types A, B, C and D was 73 (69–78.5), 76 (70.5–82), 80 (72.5–87.5) and 86.5 (76.75–95) cm (*p* < 0.0001 for all two-group comparisons, overall *p* < 0.0001, Figure 2B). The median (IQR) F index in types A, B, C and D was 5.54 (4.51–6.74), 5.88 (4.58–7.55), 6.74 (4.93–9.13) and 8.70 (6.12–11.73) kg/m^2^ (*p* < 0.0001 for all two-group comparisons, overall *p* < 0.0001, Figure 2C). The median (IQR) FLI in types A, B, C and D was 3.67 (2.19–6.81), 6.04 (3.05–13.65), 13.26 (5.35–34) and 36.41 (13.51–65.41) (*p* < 0.0001 for all two-group comparisons, overall *p* < 0.0001, Figure 2D). The median (IQR) FF index in types A, B, C and D was 14.88 (14.31–15.45), 15.04 (14.44–15.67), 15.36 (14.65–16.11) and 15.90 (15.11–16.73) kg/m^2^ (*p* < 0.0001 for all two-group comparisons, overall *p* < 0.0001, Figure 2E). The median (IQR) F-FF ratio in types A, B, C and D was 0.37 (0.31–0.45), 0.40 (0.31–0.49), 0.45 (0.34–0.57) and 0.55 (0.41–0.70) (*p* < 0.0001 for all two-group comparisons, overall *p* < 0.0001, Figure 2F).

### 3.3. The Percentage of Decreased SMM in Four Types in Men and Women

**Figure 3:**
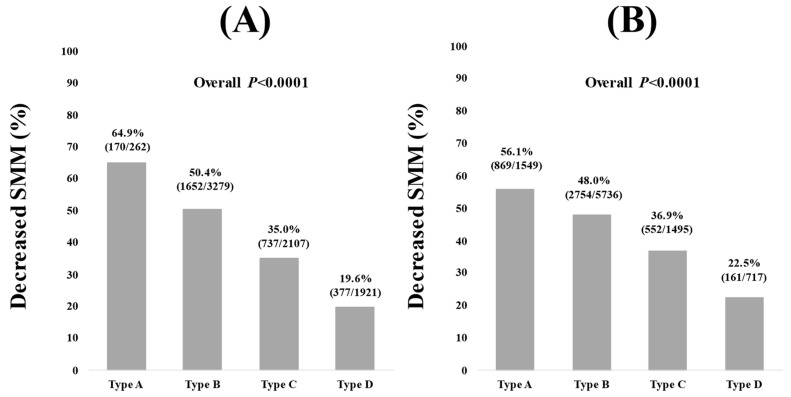
Decreased SMM among types of A, B, C and D in men (**A**) and women (**B**). Decreased SMM was defined as FF index < 18 kg/m^2^ in men and FF index < 15 kg/m^2^ in women.

The percentage of decreased SMM (FF index < 18 kg/m^2^) in types A, B, C and D in men was 64.9% (170/262), 50.4% (1652/3279), 35.0% (737/2107) and 19.6% (377/1921) (overall *p* < 0.0001, Figure 3A). The percentage of decreased SMM (FF index < 15 kg/m^2^) in types A, B, C and D in women was 56.1% (869/1549), 48.0% (2754/5736), 36.9% (552/1495) and 22.5% (161/717) (overall *p* < 0.0001, Figure 3B).

### 3.4. Body Composition-Related Parameters Among Four Types in Men with ≥50 Years and <50 Years

**Figure 4:**
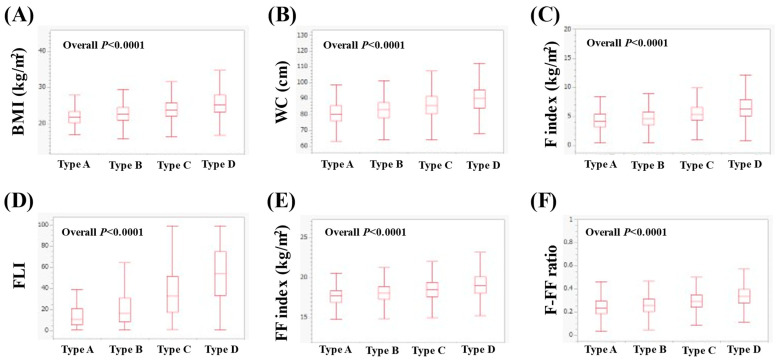
Comparison of body composition-related parameters among types A (*n* = 164), B (*n* = 2040), C (*n* = 1328) and D (*n* = 1007) in men with ≥50 years. (**A**) BMI, (**B**) WC, (**C**) F index, (**D**) FLI, (**E**) FF index and (**F**) F-FF ratio.

In men with ≥50 years (*n* = 4539), the median (IQR) BMI in types A (*n* = 164), B (*n* = 2040), C (*n* = 1328) and D (*n* = 1007) was 21.9 (20.3–23.4), 22.7 (21.1–24.5), 23.9 (22.1–25.9) and 25.3 (23.3–27.9) kg/m^2^ (*p* < 0.0001 for all two-group comparisons (except for A vs. B, *p* = 0.0002), overall *p* < 0.0001, Figure 4A). The median WC (IQR) in types A, B, C and D was 80.3 (76.1–85.5), 83 (78–87.5), 85.5 (80.5–91.5) and 90 (84–95.5) cm (*p* < 0.0001 for all two-group comparisons (except for A vs. B, *p* = 0.0004), overall *p* < 0.0001, Figure 4B). The median (IQR) F index in types A, B, C and D was 4.20 (3.22–5.42), 4.60 (3.58–5.75), 5.35 (4.34–6.59) and 6.35 (5.06–7.89) kg/m^2^ (*p* < 0.0001 for all two-group comparisons (except for A vs. B, *p* = 0.0016), overall *p* < 0.0001, Figure 4C). The median (IQR) FLI in types A, B, C and D was 11.11 (5.78–20.89), 17 (8.72–31.08), 33.08 (17.61–51.74) and 53.93 (33.3–74.88) (*p* < 0.0001 for all two-group comparisons, overall *p* < 0.0001, Figure 4D). The median (IQR) FF index in types A, B, C and D was 17.76 (16.92–18.42), 18.07 (17.29–18.89), 18.53 (17.66–19.43) and 19.06 (18.08–20.14) kg/m^2^ (*p* < 0.0001 for all two-group comparisons (except for A vs. B, *p* = 0.0002), overall *p* < 0.0001, Figure 4E). The median (IQR) F-FF ratio in types A, B, C and D was 0.23 (0.19–0.30), 0.26 (0.21–0.31), 0.29 (0.24–0.35) and 0.34 (0.28–0.40) (*p* < 0.0001 for all two-group comparisons (except for A vs. B, *p* = 0.0043), overall *p* < 0.0001, Figure 4F).

**Figure 5:**
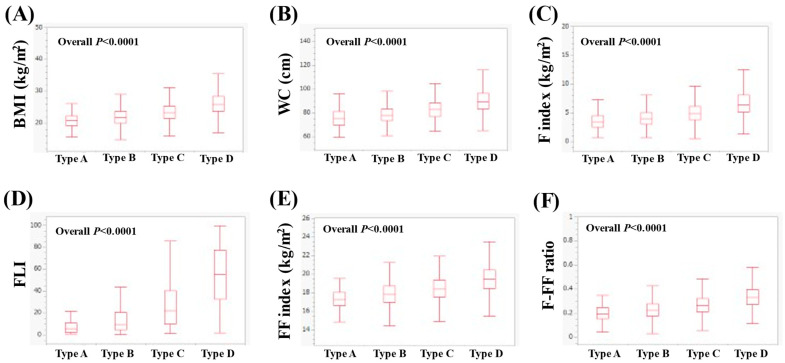
Comparison of body composition-related parameters among types of A (*n* = 98), B (*n* = 1239), C (*n* = 779) and D (*n* = 914) in men with <50 years. (**A**) BMI, (**B**) WC, (**C**) F index, (**D**) FLI, (**E**) FF index and (**F**) F-FF ratio.

In men with <50 years (*n* = 3030), the median (IQR) BMI in types A (*n* = 98), B (*n* = 1239), C (*n* = 779) and D (*n* = 914) was 20.9 (19.3–22.3), 21.8 (20.1–23.7), 23.2 (21.5–25.4) and 25.8 (23.8–28.5) kg/m^2^ (*p* < 0.0001 for all two-group comparisons (except for A vs. B, *p* = 0.0001), overall *p* < 0.0001, Figure 5A). The median WC (IQR) in types A, B, C and D was 75.5 (70–81.1), 78 (73.5–83.5), 83 (77–88.5) and 89.5 (83.5–96.5) cm (*p* < 0.0001 for all two-group comparisons (except for A vs. B, *p* = 0.0002), overall *p* < 0.0001, Figure 5B). The median (IQR) F index in types A, B, C and D was 3.44 (2.51–4.51), 3.99 (3.07–5.08), 4.85 (3.87–6.13) and 6.40 (5.15–8.11) kg/m^2^ (*p* < 0.0001 for all two-group comparisons (except for A vs. B, *p* = 0.0001), overall *p* < 0.0001, Figure 5C). The median (IQR) FLI in types A, B, C and D was 5.57 (2.43–10.90), 9.66 (4.93–20.92), 22.03 (9.97–40.35) and 55.27 (32.52–77.57) (*p* < 0.0001 for all two-group comparisons, overall *p* < 0.0001, Figure 5D). The median (IQR) FF index in types A, B, C and D was 17.25 (16.62–18.05), 17.80 (16.98–18.72), 18.41 (17.54–19.36) and 19.50 (18.46–20.48) kg/m^2^ (*p* < 0.0001 for all two-group comparisons (except for A vs. B, *p* = 0.0001), overall *p* < 0.0001, Figure 5E). The median (IQR) F-FF ratio in types A, B, C and D was 0.20 (0.16–0.25), 0.23 (0.18–0.28), 0.27 (0.21–0.32) and 0.33 (0.27–0.40) (*p* < 0.0001 for all two-group comparisons (except for A vs. B, *p* = 0.0003), overall *p* < 0.0001, Figure 5F).

### 3.5. Body Composition-Related Parameters Among Four Types in Women with ≥50 Years and <50 Years

**Figure 6:**
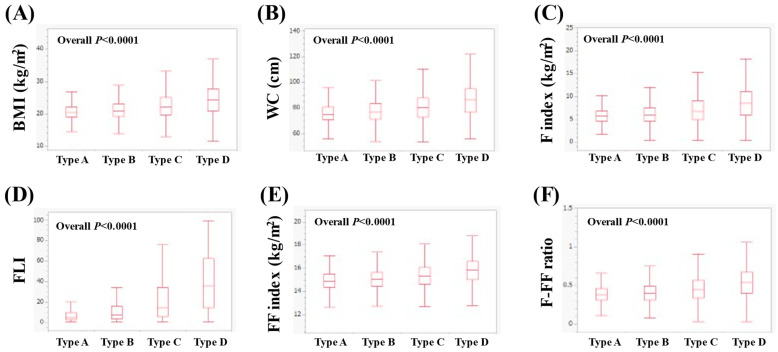
Comparison of body composition-related parameters among types A (*n* = 509), B (*n* = 3300), C (*n* = 1057) and D (*n* = 482) in women with ≥50 years. (**A**) BMI, (**B**) WC, (**C**) F index, (**D**) FLI, (**E**) FF index and (**F**) F-FF ratio.

In women with ≥50 years (*n* = 5348), the median (IQR) BMI in types A (*n* = 509), B (*n* = 3300), C (*n* = 1057) and D (*n* = 482) was 20.5 (19–22.2), 20.9 (19.2–23.1), 22.1 (19.7–25.1) and 24.3 (21.0–27.7) kg/m^2^ (*p* < 0.0001 for all two-group comparisons (except for A vs. B, *p* = 0.0016), overall *p* < 0.0001, Figure 6A). The median WC (IQR) in types A, B, C and D was 75 (71–81), 77 (71.5–83.5), 80.5 (73–88) and 86.3 (77–95) cm (*p* < 0.0001 for all two-group comparisons, overall *p* < 0.0001, Figure 6B). The median (IQR) F index in types A, B, C and D was 5.70 (4.56–6.84), 5.95 (4.60–7.55), 6.75 (4.94–9.08) and 8.52 (5.92–11.12) kg/m^2^ (*p* < 0.0001 for all two-group comparisons (except for A vs. B, *p* = 0.0103), overall *p* < 0.0001, Figure 6C). The median (IQR) FLI in types A, B, C and D was 4.78 (2.89–9.89), 7.46 (3.77–15.96), 14.43 (6.07–34.15) and 35.71 (14.55–62.58) (*p* < 0.0001 for all two-group comparisons, overall *p* < 0.0001, Figure 6D). The median (IQR) FF index in types A, B, C and D was 14.91 (14.36–15.49), 15.06 (14.47–15.65), 15.35 (14.63–16.07) and 15.84 (15.05–16.59) kg/m^2^ (*p* < 0.0001 for all two-group comparisons (except for A vs. B, *p* = 0.0005), overall *p* < 0.0001, Figure 6E). The median (IQR) F-FF ratio in types A, B, C and D was 0.38 (0.31–0.46), 0.40 (0.31–0.49), 0.45 (0.34–0.57) and 0.54 (0.40–0.67) (*p* < 0.0001 for all two-group comparisons (except for A vs. B, *p* = 0.0278), overall *p* < 0.0001, Figure 6F).

**Figure 7:**
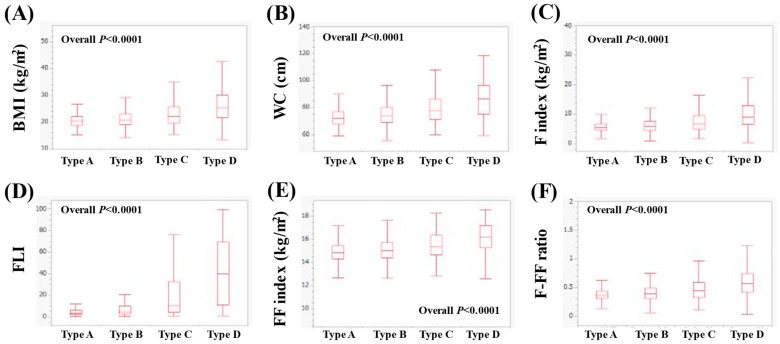
Comparison of body composition-related parameters among types of A (*n* = 1040), B (*n* = 2436), C (*n* = 438) and D (*n* = 235) in women with <50 years. (**A**) BMI, (**B**) WC, (**C**) F index, (**D**) FLI, (**E**) FF index and (**F**) F-FF ratio.

In women with <50 years (*n* = 4149), the median (IQR) BMI in types A (*n* = 1040), B (*n* = 2436), C (*n* = 438) and D (*n* = 235) was 20.3 (18.9–22), 20.8 (19–23.1), 22 (19.5–25.7) and 25.3 (21.7–30.1) kg/m^2^ (*p* < 0.0001 for all two-group comparisons, overall *p* < 0.0001, Figure 7A). The median WC (IQR) in types A, B, C and D was 72 (68–77), 74 (69–84), 78 (71.5–86) and 86.5 (75–96.5) cm (*p* < 0.0001 for all two-group comparisons, overall *p* < 0.0001, Figure 7B). The median (IQR) F index in types A, B, C and D was 5.47 (4.49–6.68), 5.79 (4.56–7.55), 6.73 (4.90–9.55) and 9.03 (6.45–12.82) kg/m^2^ (*p* < 0.0001 for all two-group comparisons, overall *p* < 0.0001, Figure 7C). The median (IQR) FLI in types A, B, C and D was 3.06 (1.95–5.85), 4.56 (2.49–9.80), 10.21 (3.92–32.85) and 39.98 (11.03–69.33) (*p* < 0.0001 for all two-group comparisons, overall *p* < 0.0001, Figure 7D). The median (IQR) FF index in types A, B, C and D was 14.87 (14.29–15.44), 15.02 (14.41–15.70), 15.38 (14.67–16.36) and 16.17 (15.27–17.22) kg/m^2^ (*p* < 0.0001 for all two-group comparisons, overall *p* < 0.0001, Figure 7E). The median (IQR) F-FF ratio in types A, B, C and D was 0.37 (0.31–0.44), 0.39 (0.31–0.49), 0.44 (0.33–0.59) and 0.57 (0.42–0.75) (*p* < 0.0001 for all two-group comparisons, overall *p* < 0.0001, Figure 7F).

## 4. Discussion

Low ALT cases are not often scrutinized in any way in daily clinical practice. Body composition, including body weight, SMM and body fat percentage, has recently attracted people’s attention not only from a cosmetic standpoint but also from the perspective of health and longevity [10,11]. With these backgrounds in mind, the purpose of this study was to identify problems in cases of low ALT in terms of body composition. The total number of male and female cases exceeded 17,000 in this study, which we believe is significant to report our study results. In our baseline data, type A/B/C/D in men and women was found in 262 (3.5%)/3279 (43.3%)/2107 (27.8%)/1921 (25.4%) and 1549 (16.3%)/5736 (60.4%)/1495 (15.7%)/717 (7.6%) (*p* < 0.0001). There were 3439 (45.4%) men and 1045 (11.0%) women who met the Japanese criteria for metabolic syndrome, WC ≥ 85 cm for men and WC ≥ 90 cm for women, respectively. It should be noted that considerable differences in baseline features are observed between men and women. The baseline characteristics may reflect the fact that Japanese BMI has been on the rise in recent years, especially in men [12].

In the summary of our investigation, the results of this study showed that BMI, WC, F-index, FLI, FF index and F-FF ratio were all significantly stratified among the four groups classified by ALT level, regardless of whether they were male or female and over or under 50 years old. Fatty liver patients improve ALT levels with weight loss [13], and ALT correlates well with metabolic syndrome [5,14,15]. BMI, WC, F index and FLI are fat-related body composition values, and their linear increase with types A, B, C and D was an easily expected result.

The FF index increases linearly with type A, B, C and D, as shown in this study. In other words, the risk of sarcopenia is the highest when ALT is less than 10, as shown in Figure 3 (the percentage of decreased SMM in types A, B, C and D in men was 64.9%, 50.4%, 35.0% and 19.6% (overall *p* < 0.0001), while those in women was 56.1%, 48.0%, 36.9% and 22.5% (overall *p* < 0.0001)). In patients with prostate cancer [16], myelodysplastic syndrome [17], chronic obstructive pulmonary disease [18] and bladder cancer [19], low ALT correlates with sarcopenia or frailty and is an adverse predictor. Even in the elderly, low ALT correlates with sarcopenia or frailty and is a poor prognostic factor [20]. That study reported a surprising 4.3- and 5.5-fold increased risk of overall mortality and cardiovascular disease-related mortality below ALT 13 IU/L, using ALT 19–23 IU/L as the reference standard [20]. These previous reports are consistent with our findings; in the case of low ALT, the assessment of SMM is mandatory. In the current study, the median age of male and female type A was 52 years and 45 years. The lower BMI in type A compared with types B, C and D may be associated with the high number of cases of reduced SMM. Low BMI is an important risk factor for sarcopenia [21].

The F-FF ratio increased linearly with types A, B, C and D in this study. This may be due to the fact that the increase in fat mass exceeds the increase in muscle mass as one progresses through types A, B, C and D. In adolescents, the FF-F ratio is associated with ALT levels [22]. An increase in the FF-F ratio was a predictor of reduced ALT, independent of age and other backgrounds in patients with non-alcoholic fatty liver disease (NAFLD), which is in line with our results [23]. An elevated F-FF ratio, rather than BMI, can be a better marker for poorer functional outcomes in pre-frail older persons [24]. An elevated F-FF ratio is significantly linked to an increased risk of NAFLD and liver fibrosis regardless of BMI [25], the severity of asthma [26], an increased risk of cardiovascular disease and its prognosis [27], insulin resistance [28] and an elevated risk of type 2 diabetes development in both non-obese and obese individuals [29]. In addition, a higher F-FF ratio is associated with natural killer cell activity decline regardless of gender [30]. The F-FF ratio has thus been demonstrated to be associated with a variety of clinical characteristics. The close correlation between the F-FF ratio and ALT appears to be of great clinical importance; low ALT may be the result of a low F-FF ratio as the extent of fat mass loss exceeds that of muscle mass loss. We previously reported a significant positive correlation between the F-FF ratio and insulin resistance (correlation coefficients (*r*) between the F-FF ratio and the Homeostasis Model Assessment of Insulin Resistance (HOMA-IR): *r* = 0.55 (*p* < 0.0001) in men (*n* = 1186) and *r* = 0.56 (*p* < 0.0001) in women (*n* = 1441)) [28]. Lower ALT levels suggest an improvement to healthy glucose metabolism.

Although this study is a single-center, retrospective study, it has the strength of being an analysis of a large number of cases, over 17,000 cases. In conclusion, with a decrease in ALT level in medical health checkups, the fat mass and F-FF ratio decrease, but a decrease in SMM cannot be overlooked. Although the lack of grip strength data makes the accurate assessment of sarcopenia difficult [31], cases with low ALT levels (especially those with ALT ≤ 10 IU/L) should be followed up with attention to sarcopenia complications.

## 5. Conclusions

It should be noted that in cases of low ALT in medical health checkups, there is a positive aspect of low fat mass and low F-FF ratio, but there is also a negative aspect of low SMM.

## Figures and Tables

**Table 1:** Baseline characteristics.

	Men (*n* = 7569)	Women (*n* = 9497)	*p* Value
Age (years)	52 (44–62)	51 (43.5–59)	<0.0001
Body mass index (kg/m^2^)	23.4 (21.4–25.7)	21.1 (19.2–23.6)	<0.0001
Waist circumference (cm)	84 (78.5–90.5)	76 (70.5–83.5)	<0.0001
Systolic blood pressure (mmHg)	121 (111–132)	112 (103–125)	<0.0001
Diastolic blood pressure (mmHg)	77 (69–85)	69 (62–78)	<0.0001
Alanine aminotransferase (IU/L)	21 (16–31)	15 (12–20)	<0.0001
Gamma-glutamyl transferase (IU/L)	30 (20–50)	17 (13–26)	<0.0001
Triglyceride (mg/dL)	97 (68–142)	69 (52–97)	<0.0001
Fasting blood glucose (mg/dL)	91 (85–99)	86 (81–92)	<0.0001
eGFR (mL/min/1.73 m^2^)	70.1 (62.0–78.7)	72.7 (64.4–81.9)	<0.0001
Fat mass index (kg/m^2^)	5.06 (3.83–6.45)	6.03 (4.67–7.94)	<0.0001
Fat-free mass index (kg/m^2^)	18.38 (17.48–19.38)	15.11 (14.48–15.77)	<0.0001
F-FF ratio	0.28 (0.22–0.34)	0.40 (0.32–0.51)	<0.0001
Fatty liver index	24.78 (10.36–49.24)	6.56 (3.11–17.23)	<0.0001

Data are expressed as median (IQR). eGFR; estimated glomerular filtration rate, F-FF ratio; fat mass to fat-free mass ratio.

## Data Availability

The data presented in this study are available on request from the corresponding author (accurately indicate status).

## Abbreviations

ALT: alanine aminotransferase; OMPU: Osaka Medical and Pharmaceutical University; F: fat, FF: fat-free; F-FF ratio: fat mass to fat-free mass ratio; SMM: skeletal muscle mass; FLI: fatty liver index; BMI: body mass index; WC: waist circumference; IQR: interquartile range; NAFLD: non-alcoholic fatty liver disease.
